# Superhydrophobic Al_2_O_3_/MMT-PDMS Coated Fabric for Self-Cleaning and Oil–Water Separation Application

**DOI:** 10.1021/acs.langmuir.3c02325

**Published:** 2023-12-05

**Authors:** S. Foorginezhad, M. Asadnia

**Affiliations:** † Department of Engineering Sciences and Mathematics, Luleå University of Technology, Energy Science, Luleå 97187, Sweden; ‡ School of Engineering, Macquarie University, Sydney, New South Wales 2109, Australia

## Abstract

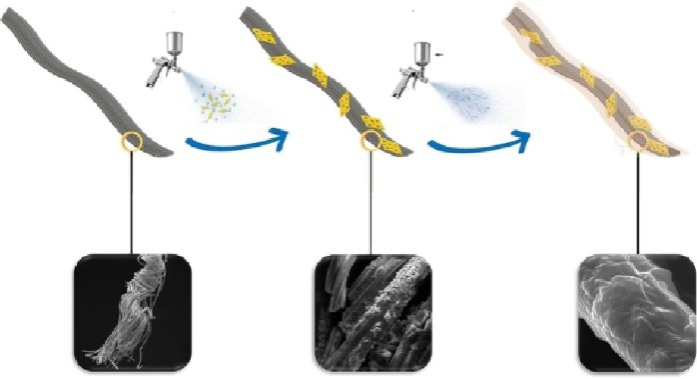

This study introduces a novel superhydrophobic coating applied to the fabric surface through spray coating of the Al_2_O_3_/MMT nanocomposite and PDMS polymer to enhance the surface roughness and reduce the surface tension, respectively. The as-prepared coating exhibits a remarkable superhydrophobic property with a water contact angle (WCA) of ∼174.6° and a water sliding angle (WSA) < 5°. Notably, the fabric demonstrates a self-cleaning property through removing dust and dirt via adhering to water droplets. Moreover, the insignificant loss of WCA (3.2 and 1%) after exposure to alkaline and acidic media for 10 days verifies the promising chemical stability of the coated layer, whereas WCA > 160° after 24 h of immersion in various organic solvents further indicates the layer resistance. Besides, the layer sustains WCA of 174.5, 172.5, and 168.45° after 1 month of air exposure, ultrasonic washing, and 50 cycles of home laundry. The mechanical resistance of the fabric was verified by maintaining a WCA of 158.73° after 200 abrasion cycles. Also, the layer exhibits thermal resistance with <4.1% of WCA loss in the temperature range of −10 to 180 °C. Additionally, the superhydrophobic coating excels in oil–water separation, achieving >99% separation efficiency for various oils. These exceptional properties position the fabric for diverse applications, including protective clothing, outdoor gear, medical textiles, and sportswear, emphasizing its versatility and novelty in the realm of superhydrophobic materials.

## 1 Introduction

The remarkable properties of superhydrophobic surfaces, such as self-cleaning, low adhesion, antibacterial, and anticorrosion characteristics,^1,2^ have driven their extensive use across diverse fields. These applications include antibacterial coatings,^3^ self-cleaning applications,^3,4^ anticorrosive surfaces,^5^ water desalination and purification techniques,^6^ catalysis processes,^7^ anti-icing surfaces,^8^ and also the textile industry.^9^ A defining trait of superhydrophobic surfaces is their exceptional ability to repel water and exhibit a high contact angle with liquid droplets. As a result, spherical droplets readily form and effortlessly roll off the surface, creating an impressive self-cleaning effect. This unique property prevents water from adhering to the surface and effectively removes dust, dirt, and contaminants, making it highly valuable in various practical applications. The self-cleaning capability of superhydrophobic surfaces has garnered significant interest in materials science and engineering, offering promising solutions for a wide range of industries.^10^ The wettability of a surface is determined by its contact surface energy and surface roughness. To acquire water-repellent property, a surface must possess a distinctive combination of low surface energy (which hinders wetting and liquid adhesion, resulting in a nonstick behavior) and surface roughness or hierarchical architectures (that enhance its water-repellent characteristics). Superhydrophobic surfaces, which are identified by a water contact angle (WCA) greater than 150° and a water sliding angle (WSA) lower than 10°, depend on the synergistic effect of low surface energy and surface roughness to exhibit their remarkable water-repellent nature.^11−15^

To meet the need for low surface energy, there has been increasing concern regarding the environmental and health risks associated with fluorinated compounds. These compounds are commonly employed in the production of superhydrophobic surfaces because of their exceptionally low surface energy. However, their widespread usage has raised significant environmental and health-related issues that require careful consideration and exploration of alternative, more sustainable approaches. In the quest for sustainable and environmentally friendly superhydrophobic surfaces, researchers have been exploring low-surface-energy materials, including waxes, carbon nanomaterials, and polydimethylsiloxane (PDMS), as alternatives to fluorinated compounds.^16^ Among the various candidates with low surface energy, PDMS stands out as a promising alternative due to its unique advantages. Notably, it offers exceptional water and chemical resistance, guaranteeing the durability and longevity of the coated layer, and low surface tension (<20 mN/m), making it highly effective in reducing the surface energy of the substrate. Moreover, its flexibility, affordability, biocompatibility, and thermal stability further contribute to its appeal as a superior choice for sustainable and long-lasting superhydrophobic surfaces.^17,18^ For instance, Zhu et al.^19^ developed superhydrophobic cotton by harnessing the synergistic effect of rGO-TiO_2_/QAS-SiO_2_ dual nanoparticles. The optimal dual-particle coating was effectively bound to the textile surface by using PDMS as a binder. The experimental findings revealed that the water contact angles of the cotton fabric could reach 154°, signifying excellent water repellency. This successful combination of functional nanoparticles and a PDMS binder demonstrates the potential of a superhydrophobic cotton fabric with enhanced properties for a wide range of applications. Hu et al.^20^ incorporated a blend of zinc oxide particles and molecular sieves within PDMS films to fabricate robust biomimetic superhydrophobic surfaces. Their efforts resulted in the development of an environmentally compatible superhydrophobic surface exhibiting an impressive water contact angle of up to 162.3° and a minimal water sliding angle of approximately 3.2°. These findings highlight the potential of such environmentally friendly superhydrophobic surfaces for various applications, benefiting from their exceptional water repellency and stability.

To increase the surface roughness, a range of materials, such as TiO_2_, ZnO, CuO, Fe_2_O_3_, and Al_2_O_3_ particles, are utilized to enhance the surface roughness.^21^ A common issue encountered in many coatings is their susceptibility to detachment from the substrate surface when they are exposed to the scouring action of running water. This is mainly attributed to the low adhesion strength between the coating and the substrate. As a result, when exposed to harsh conditions, such as hot water or corrosive solutions, these surfaces tend to lose their super hydrophobicity. These drawbacks present significant challenges to their practical application, especially in large-scale industrial oily wastewater treatment. Hence, there is a pressing demand to develop an efficient and straightforward method to produce stable and durable superhydrophobic coatings for effective oil/water separation. Addressing these issues would greatly enhance the viability and reliability of such coatings in real-world applications.^22^

The most common methods that have been employed to introduce hydrophobicity onto various substrates include lithography,^23^ chemical vapor deposition (CVD),^24^ electrospinning,^25^ template methods,^26^ sol–gel method,^27^ and spray coating.^28^ Each of these methods has its own set of advantages and disadvantages, which are detailed in Table 1. As can be seen from Table 1, the most common disadvantage encountered across different methods used for the fabrication of superhydrophobic surfaces is the complexity and cost associated with the process. Also, most of the techniques utilized require special equipment, controlled environments, or multiple processing steps, which can increase the complexity and overall cost of fabrication. Among them, spray coating offers several advantages over other methods, including a simple and cost-effective technique that can be easily scaled up for large-scale production. As a noncontact method, spray coating minimizes the risk of damaging delicate substrates and allows for uniform and controlled deposition of coating materials onto complex-shaped substrates, including fabrics. It offers versatility in terms of coating thickness, surface roughness, and ability to incorporate various functional additives.

**Table 1:** Most Common Methods Used for the Fabrication of Superhydrophobic Surfaces

method	advantages	disadvantages	ref
templating technique	low cost, reusability, controllability	complex, only applicable to a limited number of materials, time consuming	(35)
sol–gel process	applicable to various substrates with homogeneous coating	regular monitoring of the process, shrinkage, increase in carbon content while using organic reagents during the preparative step, formation of crack while drying	(36)
layer-by-layer deposition	molecularly precise control of the film thickness	hydrophilization is often necessary, some additional steps such as the incorporation of nanoparticles are needed	(37)
lithography	useful method for fabricating rough surfaces with regular structures	complicated to form superhydrophobic surfaces especially for large-scale surfaces, costly	(38)
chemical etching	suitable for surface roughness on polycrystalline metals, simple technique	formation of irregular surface structures	(38)
chemical vapor deposition (CVD)	applicable to various types of substrates	costly, deposition rate is very slow	(39)
spray coating	simple and convenient method, suitable method for making products with complex structure, wide application scope, some special reagents can be added to increase the wear resistance of the surface	thickness control is challenging	(40)

Textiles hold significant importance among the various substrates explored for the fabrication of superhydrophobic surfaces. This is primarily due to their extensive range of applications in human life and various industries, including food packaging, medical devices, garment production, sports equipment, wastewater treatment, and separation.^29−32^ There is a growing demand for the advancement of textile technology to cater to specialized, such as antibacterial, flame-resistant, UV-protective, and hydrophobic, applications. These requirements arise from the increasing needs of properties that traditional textiles alone cannot adequately fulfill.^1,33,34^ Introducing a superhydrophobic property to the surface of textiles can be a promising approach to prevent moisture absorption, inhibit mold growth, and minimize degradation caused by exposure to water and other liquids. This property also reduces the possibility of staining, resulting in less frequent washing and maintenance, ultimately making it more convenient for everyday usage.

Clay-based coating demonstrates a significant advantage over the reported materials such as metal nanoparticles in terms of corrosion resistance, mechanical stability, and high-temperature resistance for fabricating superhydrophobic coatings.^22^ Montmorillonite (MMT) is a natural clay mineral with a unique structure comprising an insoluble large layer with weakly bound cations residing in the interlayer space. This hydrated aluminum silicate possesses a layer of aluminum oxide sandwiched between two layers of silicon oxide. The layers are held together by weak van der Waals forces, which allow them to easily slide over one another, giving montmorillonite its unique swelling and absorbent properties. This clay mineral is known for its high cation exchange capacity, surface area, and ability to interact with water and other molecules, making it valuable in various applications, including as a catalyst, as an adsorbent, and in the fabrication of superhydrophobic coatings. In the quest to fabricate a superhydrophobic coating, this paper focuses on enhancing the surface roughness and reducing the surface tension. To achieve the hierarchical micro/nano roughness, the MMT mineral was utilized as the substrate to incorporate Al_2_O_3_ nanoparticles into the layers, and the as-prepared nanocomposite was spray-coated onto the fabric surface. Subsequently, to reduce the surface energy, a layer of PDMS polymer was spray-coated onto the fabric. The resulting Al_2_O_3_/MMT-PDMS coated fabric underwent comprehensive characterization using WCA and WSA measurements, evaluation of chemical and thermal stability, assessment of mechanical abrasion resistance and washing durability, as well as further analyses using SEM, EDX, PSA, and FTIR. Additionally, owing to its high water repellency, the superhydrophobic fabric was subjected to various oil–water mixture separations, and the separation efficiency was thoroughly evaluated. Regarding the results, this study introduces novel contributions to the existing knowledge about superhydrophobic and self-cleaning coatings. By providing an efficient and sustainable approach for the fabrication of superhydrophobic coatings, this work adds significant value to the field and addresses the key challenges associated with conventional methods.

## 2 Experimental Section

### 2.1 Materials and Methods

#### 2.1.1 Materials

Polydimethylsiloxane (OH terminated PDMS, 750 cSt % viscosity), dibutyltin dilaurate (DBTDL, 95% purity), tetraethyl orthosilicate (TEOS, 98% purity), *n*-heptane (99% purity), sodium chloride (NaCl), aluminum nitrate (Al(NO_3_)_3_·9H_2_O, 95%), ammonium bicarbonate (NH_4_HCO_3_, 98%), dodecyltrimethoxysilane (DTMS, 99% purity), dichloromethane (DCM), dimethylformamide (DMF), hexane, toluene, ethyl acetate (EA), ethylene glycol (EG), petroleum ether, kerosene, paraffin, and acetone were purchased from Sigma-Aldrich Co. Montmorillonite (MMT) was purchased from Nanosany Co. (Mashhad, Iran). Sunflower oil was purchased from a local market. Deionized water was used for aqueous solution preparation. All of the reagents were utilized as received without further purification.

#### 2.1.2 Preparation of the Al_2_O_3_/MMT Nanocomposite

##### 2.1.2.1 MMT Ion Exchange

Cation exchange was achieved through the dispersion of 50 g/L MMT suspension in 400 mL NaCl (1 mol/L) followed by 3 h stirring at 500 rpm at 70 °C.^41−43^ Then, the suspension was washed with deionized water and centrifuged several times. Na-MMT was prepared by the dispersion of 10 g/L Na-MMT suspension by an ultrasonic dispersion instrument with 450 W power for 5 min.^41^

##### 2.1.2.2 Exfoliation of Ion Exchanged MMT

According to the overall procedure reported in a previous study,^44^ Na-MMT was dispersed in ultrapure water with 5 wt % concentration. Subsequently, the exfoliation of ion-exchanged MMTs was carried out using an ultrasonic dispersion instrument (MH S3 220, Soltec Co., Italy) at 40% power and a strength of 450 W/cm^2^ for 10 min until the MMTs were exfoliated into monolayers.

##### 2.1.2.3 Nanocomposite Preparation

**Figure 1:**
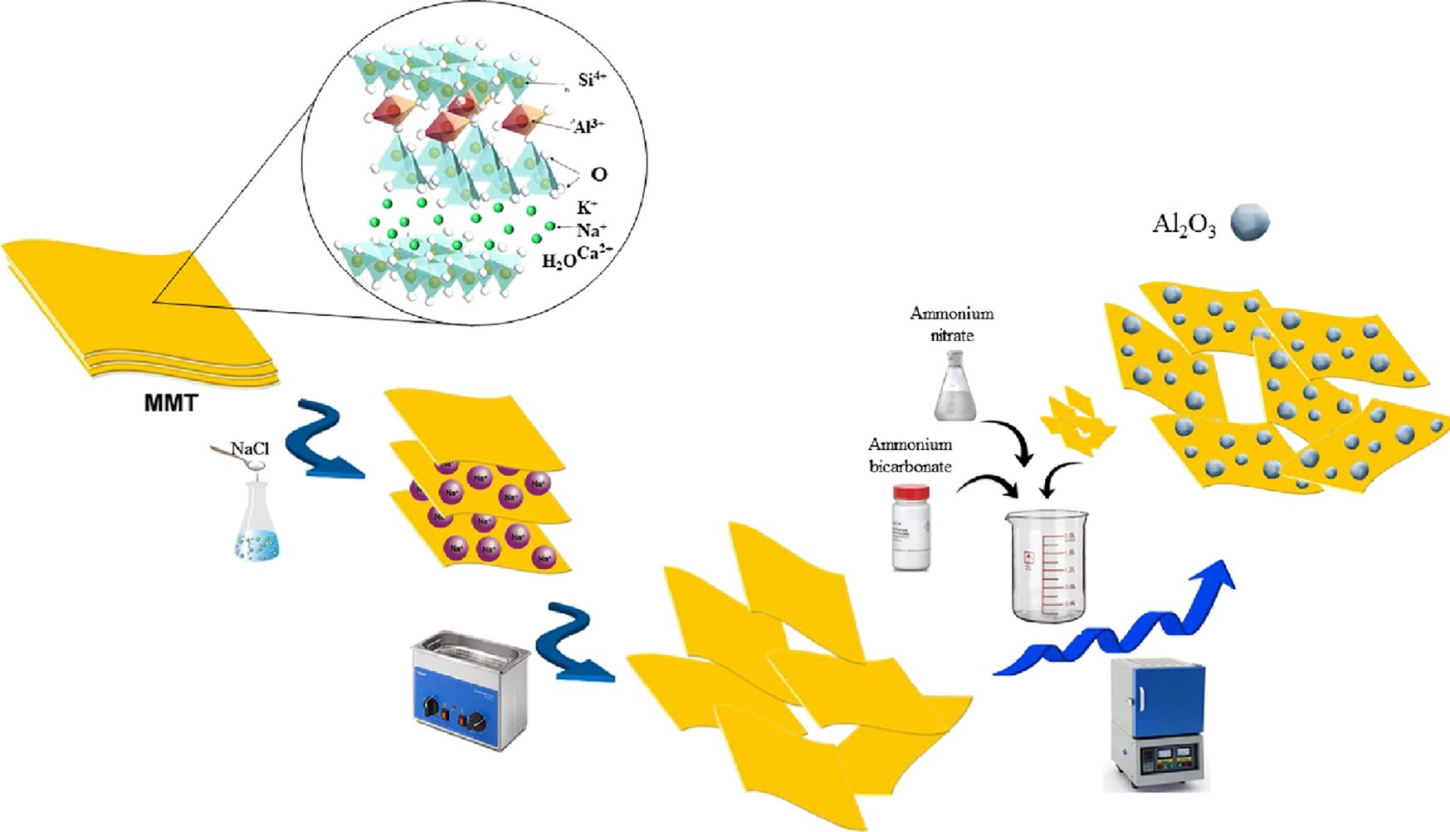
Preparation procedure of the Al_2_O_3_/MMT nanocomposite.

The Al_2_O_3_/MMT nanocomposite was synthesized through the overall procedure reported in previous studies.^43,45^ To this end, 0.04 mol/L aluminum nitrate and 0.076 mol/L ammonium bicarbonate aqueous solutions were first prepared. On the other hand, 30 mL of deionized water was added to a round-bottom flask followed by adding 2g of Na-MMT and stirring while heating at 70 °C. Then, aluminum nitrate and ammonium bicarbonate solutions were added dropwise to the Na-MMT suspension simultaneously. The final mixture was stirred at 70 °C for 3 h, whereas the pH of the solution was adjusted to 7.5–8.5 using NaOH or HNO_3_. Finally, the precipitate was filtered and washed with hot water, acetone, and ethanol to remove excess Na^+^ and unreacted chemicals. After drying, the sample was calcined at 550 °C for 5 h and 2 °C/min rate (Figure 1 depicts the preparation procedure of the nanocomposite).

#### 2.1.3 Preparation of the PDMS Solution

According to our previous studies, the PDMS solution was prepared by the modified procedure reported in previous studies.^21,46,47^ Precisely, 1.5 g of PDMS, 0.3 g of DBTDL (catalyst), and 0.3 g of TEOS (hardener, cross-linking agent) were mixed in 15 mL of *n*-heptane for 1 h.

#### 2.1.4 Preparation of Superhydrophobic Surfaces

The solution for the first layer deposition was prepared using a modified procedure described in the study conducted by Ji et al.^48^ Precisely, 0.5 g of the Al_2_O_3_/MMT nanocomposite was dispersed in 10 mL of ethanol using ultrasonic agitation for 30 min. Subsequently, 0.5 g of DTMS was combined with 10 mL of ethanol and 5 mL of 0.1 mol/L acetic acid and subjected to ultrasonic agitation for 30 min to ensure complete mixing (the as-prepared solution is denoted as A).

Fabric pieces (1 × 1 cm^2^) were subjected to washing with ethanol and acetone followed by drying at 60 °C for 1 h. Subsequently, a 1.5 mL volume of solution A was sprayed over the fabric surface using a glass vaporizer and dried again at 60 °C for 1 h. As per the findings of the Srinivasan et al. study,^49^ variations in the spraying distance within the range of 20–30 cm did not lead to any noticeable changes in the microstructure of the coating.

**Figure 2:**
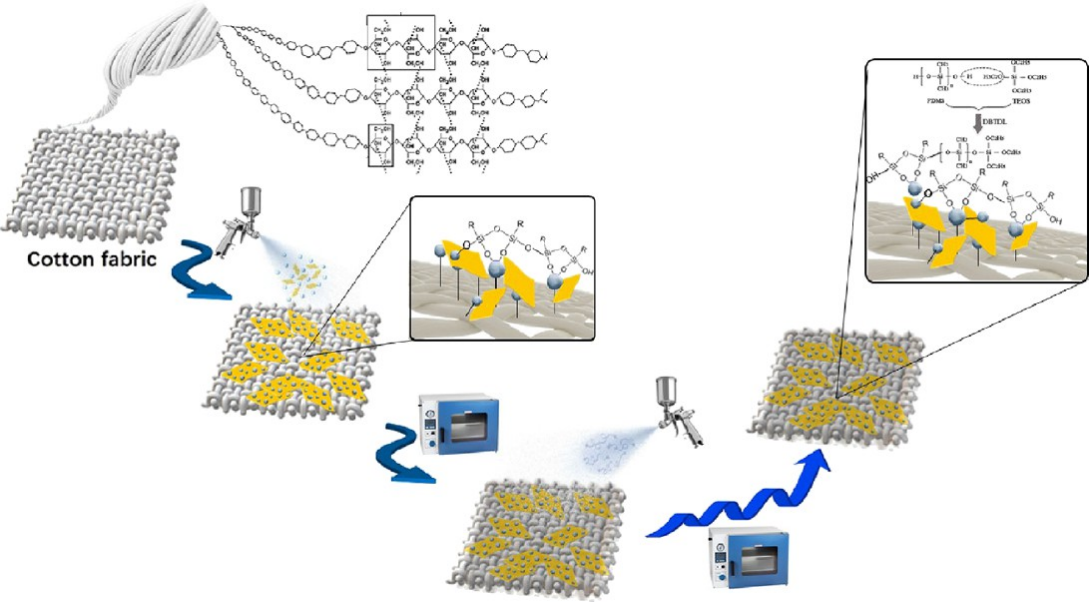
Preparation procedure of the Al_2_O_3_/MMT-PDMS coated superhydrophobic fabric.

Based on the optimal amounts determined in our previous study, a 1.5 mL volume of PDMS solution was sprayed onto the fabric surface and subsequently dried at 60 °C.^47^ (Figure 2 depicts the preparation procedure of the superhydrophobic fabric).

### 2.2 Characterization Methods

Scanning electron microscopy (SEM) equipped with an energy-dispersive X-ray (EDX) apparatus (VEGA3 TESCAN) was used to evaluate the surface texture and elemental analysis of pristine and coated samples.

The chemical composition of the prepared nanocomposite and fabric (before and after modification) was studied using attenuated total reflectance-Fourier transform infrared (ATR-FTIR) spectroscopy (AVATAR, Thermo Co., USA, spectrometer).

Dynamic light scattering (DLS) particle size analysis was performed by using a particle size analyzer (JAPA Horiba LB550) to determine the particle size distribution (PSD).

The apparent water contact angle (WCA) of a droplet on the uncoated and coated surfaces was evaluated by placing 15 μL water droplets on different parts of the surface and capturing the image using a digital microscope connected to the computer (USB, 500X), and then the contact angles were measured in the ImageJ software.

The chemical stability of the coated surfaces was studied via immersion in strong alkaline (NaOH, pH 12) and acidic (H_2_SO_4_, pH 2) media up to 10 days. At each time interval, the fabric sample was thoroughly washed with water, dried, and subjected to WCA measurement. The durability of the coatings was investigated by ultrasonic washing, home laundering, and exposure to the open air for a long time. In terms of home laundering, the fabric was washed using water and a conventional detergent for 45 min and in 50 cycles. After each cycle, the fabric was dried and subjected to WCA measurement.

The coated surface was positioned on a rotating plate to measure the sliding angle. A water droplet was then carefully placed on the surface, and the tilt angle of the plate was slowly increased from 0°. The rate of the tilt increment was approximately 0.5°/s. The experiment was repeated at least five times, each time on different surface locations, to ensure the reliability and accuracy of the measurements.

Mechanical abrasion was assessed using an 800 SiC mesh. To this end, the coated sample was placed on the mesh, and a 200 g weight was placed on that. Then, the fabric and weight were dragged through a 20 cm distance in a back-and-forth direction for 200 cycles. WCA was measured after each cycle.

The thermal resistance of the coating was evaluated through exposing the coated fabric to different temperatures from −10 to 180 °C for 12 h. After each treatment, fabric samples were subjected to the WCA measurement.

Optical micrographs of the oil–water emulsions were acquired by using a digital camera connected to an optical microscope (Olympus, BX-51M, USA) both before and after the separation process.

## 3 Results and Discussion

### 3.1 Particle Size Distribution of the Al_2_O_3_/MMT Nanocomposite

**Figure 3:**
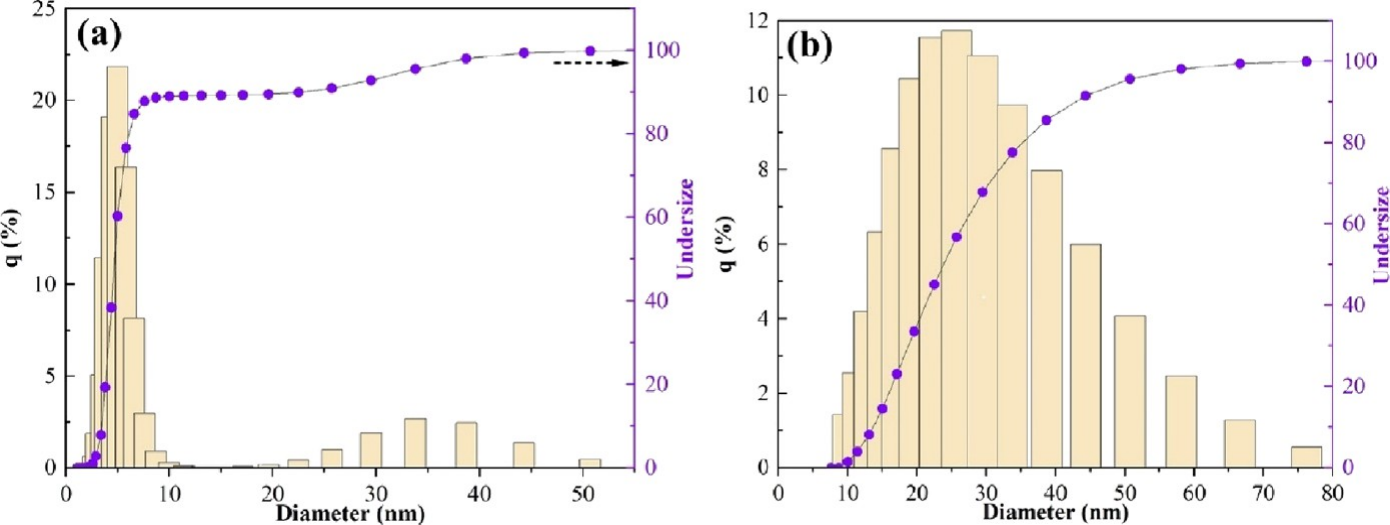
Particle size distribution of the (a) initial MMT and (b) Al_2_O_3_/MMT nanocomposite.

The particle size distribution characterizes how the particles with different sizes are distributed and is evaluated based on their diameter or equivalent diameter. Based on cumulative and density distributions, the particle size distribution of pristine MMT and Al_2_O_3_/MMT nanocomposite is delineated in Figure 3a,b. Regarding Figure 3a, the particle size of MMT is in the 2–58.1 nm range with a 7.7 nm mean size, whereas ∼90% of particles are <22.5 nm. After the Al_2_O_3_ impregnation, the particle size was shifted to the 10–76.2 nm range with a 26.3 nm mean, and ∼90% of particles showed <44.3 nm size. As can be seen, the incorporation of Al_2_O_3_ particles to the MMT surface led to an increase in the overall particle size, which exhibits good compatibility with a previous study.^43^

### 3.2 FTIR Analysis

**Figure 4:**
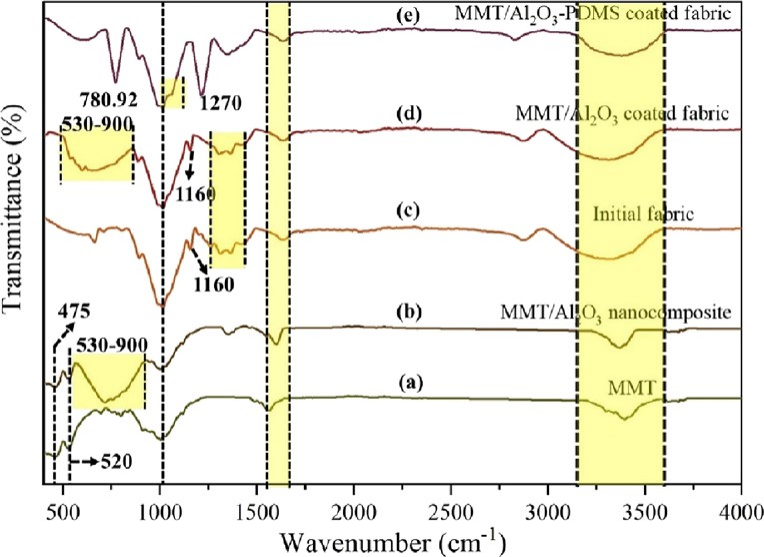
FTIR spectra of (a) MMT, (b) Al_2_O_3_/MMT nanocomposite, (c) initial fabric, (d) Al_2_O_3_/MMT coated fabric, and (e) Al_2_O_3_/MMT-PDMS coated fabric.

To confirm the chemical composition, bonding characteristics, and successful coating of the nanocomposite over the fabric surface, FTIR analysis of MMT, Al_2_O_3_/MMT nanocomposite, and pristine/coated fabric was performed. The FTIR spectra are presented in Figure 4, providing valuable insights into these aspects. In all samples, a broad peak within the range of 3450–3625 cm^–1^ can be attributed to the stretching vibration of surface OH groups,^50,51^ and in the case of MMT, this broad peak originates from the stretching vibration of structural OH groups^52^ in addition to adsorbed moisture. Additionally, a smaller peak around ∼1645 cm^–1^ indicates the bending vibration of adsorbed water.^53^

Figure 4a depicts the FTIR spectrum of MMT. The 910 and 850 cm^–1^ peaks can be related to the bending vibration of AlAlOH and AlMgOH, respectively.^52^ Furthermore, peaks at 1030.5, 520, and 475 cm^–1^ originate from the stretching vibration of Si–O, bending vibration of Al–O–Si, and bending vibration of Si–O–Si, respectively.^52^ These peaks provide valuable information about the specific chemical bonds and molecular vibrations present in the MMT structure. Figure 4 confirms the successful incorporation of Al_2_O_3_ into the MMT structure. The broad peak at 530–900 cm^–1^ verifies the presence of Al–O–Al bonds with stretching vibration.^54,55^

The FTIR spectrum of the initial fabric is delineated in Figure 4c. The absorption peak at ∼2800 cm^–1^ corresponds to the C–H stretching vibration,^56^ whereas peaks that appeared at 1370.2 and 1305.41 cm^–1^ depict the bending and wagging of C–H groups,^57^ respectively. Moreover, C–O–C stretching absorption is confirmed by the observed peaks located at 1160 and 1021.13 cm^–1^.^56^ In Figure 4d, it is evident that the main characteristic peaks of the fabric are preserved. However, with the incorporation of the Al_2_O_3_/MMT nanocomposite into the fabric, a broad peak is observed in the 530–900 cm^–1^ range, which closely aligns with the FTIR spectra of the Al_2_O_3_/MMT nanocomposite. This observation suggests that the addition of the nanocomposite has influenced the spectral features of the fabric, likely due to the presence and interaction of the nanocomposite and the fabric surface. DTMS undergoes hydrolysis in the solution and reacts with the hydroxyl groups present on the surface of the nanocomposite and fabric. During this process, the methyl group in the DTMS molecule will be replaced by hydroxyl groups. Consequently, a dehydration condensation reaction may occur between the active −OH groups originating from both DTMS and the surfaces of the nanocomposite and fabric. The absorption peaks observed at 2914 and 2842 cm^–1^ are characteristic of the – (CH_2_)_*n*_– chain present in DTMS. Additionally, the bands appearing at 1108 and 1035 cm^–1^ can be attributed to the Si–O–Si groups originating from the DTMS molecules.^48^ These peaks may overlap with the strong absorption of OH and C–O–C peaks from the fabric as well as the Al–O–Si and Si–O–Si peaks of the nanocomposite.

Finally, Figure 4e depicts the Al_2_O_3_/MMT-PDMS coated fabric. Two sharp peaks appeared at 1270 and 780.92 cm^–1^ arise from −CH_3_ deformation vibration and Si–C stretching vibration.^58^ The absorption peaks observed in the range 1000–1135 cm^–1^ can be attributed to the Si–O–Si network. These peaks are overlapped by the C–O bending modes typically found in cellulose.^59^ The presence of these peaks indicates the successful deposition of the PDMS coating onto the fabric surface as well as the incorporation of the Al_2_O_3_/MMT nanocomposite.

### 3.3 Surface Morphology

**Figure 5:**
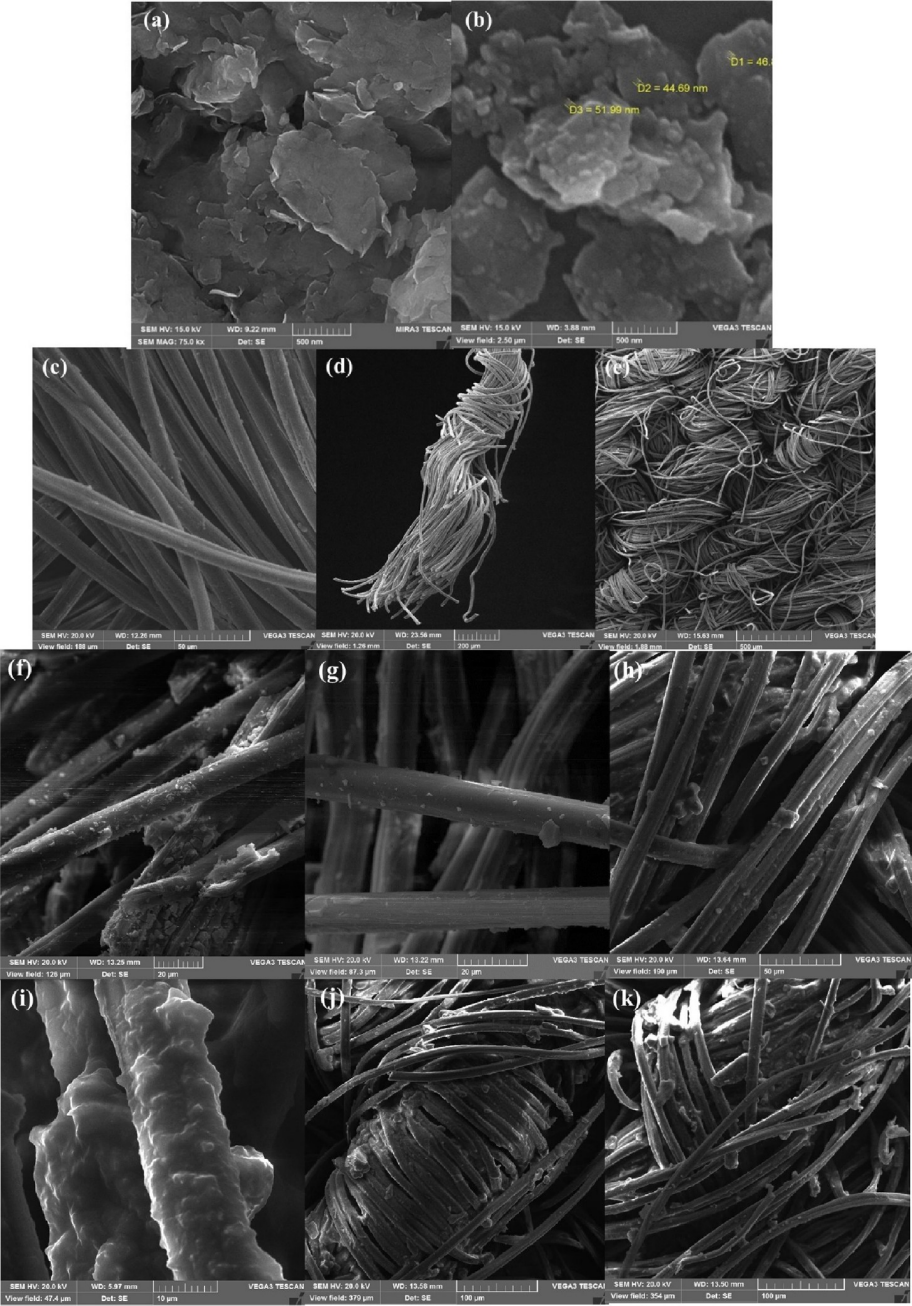
SEM micrographs of (a) MMT, (b) Al_2_O_3_/MMT nanocomposite, (c–e) pristine, (f–h) Al_2_O_3_/MMT nanocomposite coated, and (i-k) Al_2_O_3_/MMT nanocomposite-PDMS coated fabrics.

The surface morphology of MMT, Al_2_O_3_/MMT nanocomposite, and pristine/coated fabric that refers to the visual appearance and structure of the materials’ surface at a microscopic level, including the topography, texture, and features present on the surface as well as fiber size, were evaluated with different magnifications using SEM micrographs, and the results are shown in Figures 5a–i.

#### 3.3.1 MMT and Al_2_O_3_/MMT Nanocomposite

Figure 5a demonstrates the relatively consistent morphology of the MMT clay characterized by predominantly flat layers of several microns in length, which is in good accordance with previous studies.^60^ The morphology of the Al_2_O_3_/MMT nanocomposite is delineated in Figure 5b. The formation of Al_2_O_3_ particles with <100 nm size over the thin, plate-like structures with a layered arrangement of MMT can be seen. Incorporation and dispersion of Al_2_O_3_ nanoparticles onto the MMT surface resulted in surface irregularities. The Al_2_O_3_ nanoparticles, acting as protrusions on the MMT surface, introduce micro/nanosized roughness. The as-prepared Al_2_O_3_/MMT nanocomposite has been previously synthesized in our study and used for water treatment application after further modifications.^43^

#### 3.3.2 Pristine Fabric

Representative SEM micrographs of the initial fabric are delineated in Figures 5c–e. The texture of the woven cotton fabric shows the smooth surface of the fibers. It can be seen that the average diameter of the uncoated fibers was ∼12.5 μm and the three-dimensional microfibers were well-oriented with good compatibility with previous studies.^21,56,61^ Images with varying magnifications (ranging from 50 to 500 μm) have been selected to show the fibers and highlight the texture of the fabric at different scales.

#### 3.3.3 Al_2_O_3_/MMT Nanocomposite Coated Fabric

SEM micrographs of the Al_2_O_3_/MMT nanocomposite-coated fabric are shown in Figure 5f–h. It can be seen that nano, submicron, and microparticles were spread over the surface of the fibers. The random dispersion of the nanocomposite in addition to the formation of clusters and agglomerated particles over the smooth fibers provided hierarchical (nano- and microscale) roughness as an imperative factor in making a superhydrophobic surface. The surface morphology of the Al_2_O_3_/MMT nanocomposite coated cotton showed a good compatibility with previous studies, including TiO_2_/spacer succinate films grafted onto nylon,^62^ SiO_2_-aerogel/polyurethane coated fabric,^63^ AgBr-TiO_2_/OV-POSS coated fabric,^64^ TiO_2_/OV-POSS/PDMS coated fabric,^21^ and nano-TiO_2_ coated cotton yarn.^65^ It is suggested that the presence of voids between the cotton fibers and within the incorporated particles plays a crucial role in the formation of the Cassie–Baxter model. These voids result in the creation of air pockets within the grooves of the rough surface, preventing direct contact between the droplet and the surface.^66^ Images at varying magnifications (ranging from 20 to 50 μm) delineate the incorporation of the Al_2_O_3_/MMT nanocomposite and agglomeration of these particles over the fibers.

#### 3.3.4 Al_2_O_3_/MMT-PDMS Coated Fabric

The PDMS polymer was sprayed over the surface to chemically decrease the surface tension, and the ultimate morphology of the as-prepared fabric was considered using SEM micrographs (Figure 5i–k). From Figure 5i, it can be seen that the fibers covered the PDMS layer. Furthermore, the PDMS coating displayed uniformity as it formed a thin layer over the individual fibers without any signs of agglomeration or sticking. Also, the surface of the fibers exhibited protrusions, indicating the presence of Al_2_O_3_/MMT nanocomposites on their surface. Regarding Figure 5i–k, the average thickness of the fibers was increased up to ∼14.2 μm (∼13.6% increase in thickness in the average fiber diameter after PDMS coating). Upon visual inspection, there are no apparent differences in the appearance and woven structures between initial and coated fibers. However, regarding SEM micrographs with high magnification, it becomes evident that the roughness of the individual fiber in the coated fabric increased through the formation of micro/nanostructures and further polymer coating applied to the surface.^67^ Overall, the comprehensive coverage of fibers coated with the Al_2_O_3_/MMT nanocomposite and a subsequent PDMS layer, effective coating on individual fibers, well-dispersed and adherent coating, and the surface roughness are delineated in micrographs at different magnifications (10 to 100 μm).

### 3.4 Surface Elemental Composition

**Figure 6:**
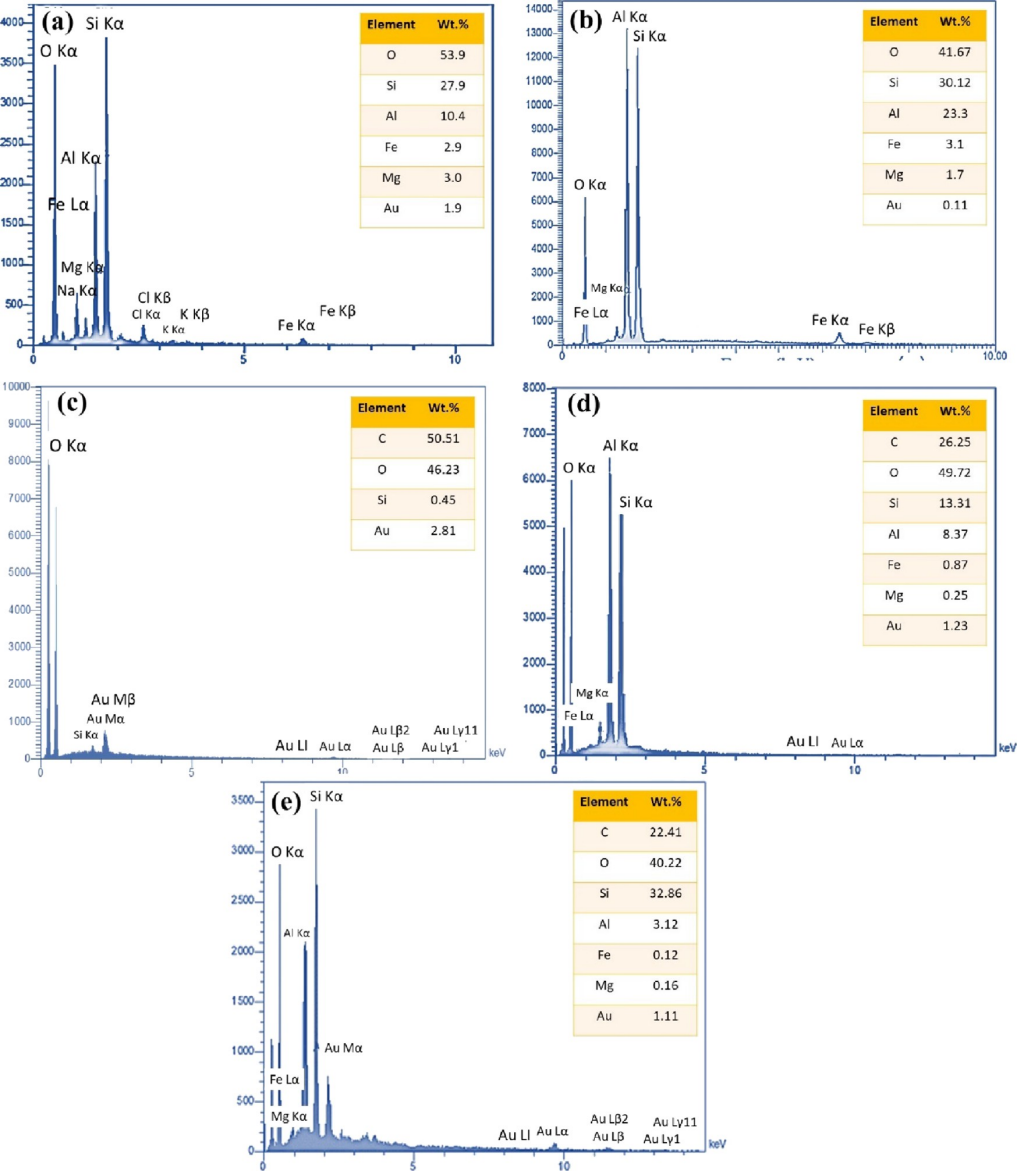
EDX results of (a) MMT, (b) Al_2_O_3_/MMT nanocomposite, (c) pristine fabric, (d) Al_2_O_3_/MMT nanocomposite coated fabric, and (e) Al_2_O_3_/MMT-PDMS coated fabric.

Figures 6 depicts the EDS analysis of MMT, Al_2_O_3_/MMT nanocomposite, and fabrics (pristine and coated). Using EDS, it is possible to determine the relative abundance and distribution of different elements, providing insights into the modifications resulting from the incorporation of the Al_2_O_3_/MMT nanocomposite and PDMS polymer onto the fabric surface. It enables the identification and quantification of elements present in each material, allowing for a comprehensive understanding of their elemental composition. This analysis helps elucidate the changes in elemental content and distribution induced by adding the Al_2_O_3_/MMT nanocomposite and PDMS polymer to the fabric surface, facilitating a deeper understanding of the modified fabric’s composition and properties.

Figure 6a illustrates the elemental composition of MMT, revealing the presence of the elements O, Si, Al, Fe, and Mg. These findings align well with previous studies,^68−70^ indicating compatibility with the reported MMT composition. Specifically, the analysis confirms that O (53.9 wt %) and Si (27.9 wt %) are the predominant elements in MMT, further verifying the elemental makeup of MMT. Figure 6b presents the distribution of various elements in the Al_2_O_3_/MMT nanocomposite. The analysis shows the presence of Al among the detected elements, which confirms the successful incorporation of Al_2_O_3_ into the MMT matrix. This observation substantiates the integration of Al_2_O_3_ nanoparticles within the MMT structure, as depicted in the elemental distribution of the nanocomposite.

Regarding Figure 6c, the elemental composition analysis of the pristine fabric reveals that it is primarily composed of C at 50.51 wt % and O at 46.23 wt %, with a minor presence of Si at 0.45 wt %. These results are consistent with previous findings and further confirm the fabric’s cellulosic nature.^71^Figure 6 provides evidence for incorporating the Al_2_O_3_/MMT nanocomposite into the fabric surface. The analysis shows that 8.37 wt % of Al has been introduced to the fabric surface accompanied by an increase in Si and O content, reaching 13.31 and 49.72 wt %, respectively. These results confirm the successful addition of the Al_2_O_3_/MMT nanocomposite to the fabric surface, as depicted in the element distribution analysis. Figure 6e confirms the subsequent coating of PDMS onto the surface. The increase in Si content up to 32.86 wt % indicates the formation of a PDMS coating on the surface. Concurrently, the Al content decreases to 3.12 wt %, suggesting that it is covered by the PDMS layer. Additionally, the presence of O and C can be attributed to the Si–O–Si bonds and CH_3_ groups of PDMS on the surface, respectively. These observations substantiate the successful deposition of PDMS onto the surface, as indicated by the corresponding elemental composition analysis.

### 3.5 Wettability

The wettability of a superhydrophobic surface refers to its ability to repel liquids, typically water, and maintain a high WCA along with low water adhesion. The optimum amount of the nanocomposite and PDMS that had to be coated over the surface was determined in our previous study. Thus, 1.5 mL of the Al_2_O_3_/MMT nanocomposite and PDMS were sprayed over the fabric, and the results are summarized in Table 2. As can be seen, the initial cotton is superhydrophilic, and water droplet instantly spread over and was absorbed by the surface, which can be related to the hydrophilic OH groups of the surface.^57^ To evaluate the affinity of the Al_2_O_3_/MMT nanocomposite toward water, 1.5 mL of solution A was sprayed over the fabric, and results showed that because of the hydrophilic nature of the Al_2_O_3_/MMT nanocomposite^72^ coating, the water droplet was immediately adsorbed by the fabric. After cross-linking and polymerization of PDMS, the water droplet was stable for several seconds and then started to spread over the surface, which was in good accordance with the previous study.^21,47^ Regarding the hydrophobic nature of PDMS, water adsorption by the PDMS-coated fabric can be attributed to smooth fibers with plentiful gaps in between.^21,73^ An exorbitant increase up to 174.6° in WCA was observed by coating a double layer of 1.5 mL of Al_2_O_3_/MMT and PDMS to the fabric surface. The high contact angle indicates that the liquid droplets on the surface are highly rounded and tend to bead up rather than spread out. The formation of a composite solid–liquid–air interface effectively hinders the penetration of water droplets to the fabric surface, and air pockets trapped between the surface roughness reduce the interaction between water and the coated surface.^74^ The promising superhydrophobicity can be attributed to the combination of the low surface energy of PDMS and the micro/nano rough structure formed by the integration of the PDMS polymer coating and Al_2_O_3_/MMT nanocomposite. The rough texture characterized by micro/nano bumps on the fabric’s surface closely resembled the natural structure found on a lotus leaf. This structural similarity contributes to the fabric’s ability to show the unique lotus effect.^75^ Similar to the behavior of the water droplet placed on the as-prepared superhydrophobic surface represented in Table 2, Video S1 in the Supporting Information verifies the superhydrophobic behavior of the Al_2_O_3_/MMT-PDMS coated fabric in contact with the water droplet.

**Table 2:**
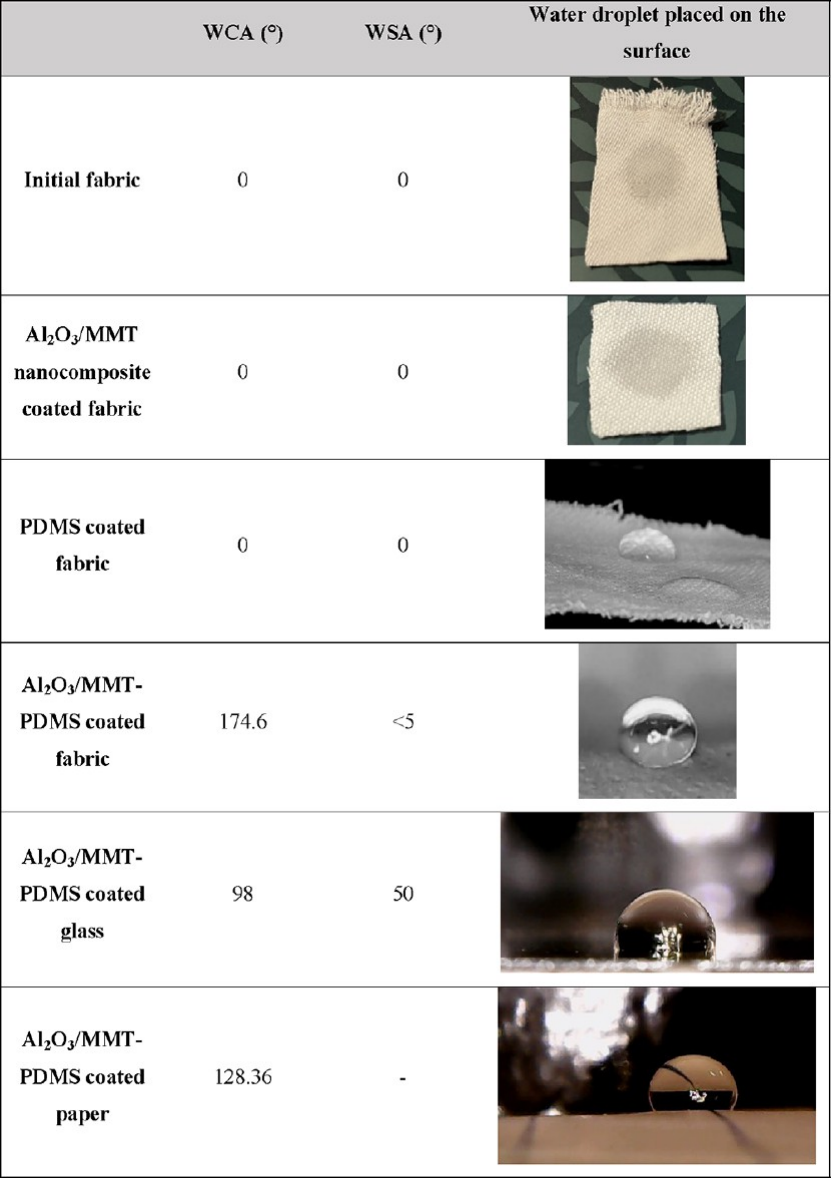
Water Droplet Behavior on Pristine and Coated Fabric, Glass, and Paper

The Al_2_O_3_/MMT-PDMS double layer was also coated on paper and glass, and results revealed WCA of 128.36 and 98°, respectively. The lower WCA of the coated paper and glass compared to the coated fabric can be attributed to the smoother surface texture. In terms of sliding angle, water droplets started to slide when the Al_2_O_3_/MMT-PDMS coated fabric was tilted <5°, which depicts that water molecules did not establish a strong bond with the surface, whereas ∼50° tilting angle was required for water droplets placed on the coated glass to slide. On the other hand, water droplets did not slide off the coated paper. Overall, the applied double-layered nanocomposite/polymer coating resulted in the formation of superhydrophobic fabric, hydrophobic paper, and hydrophobic glass.

### 3.6 Self-Cleaning and Oil–Water Separation Efficiency

**Figure 7:**
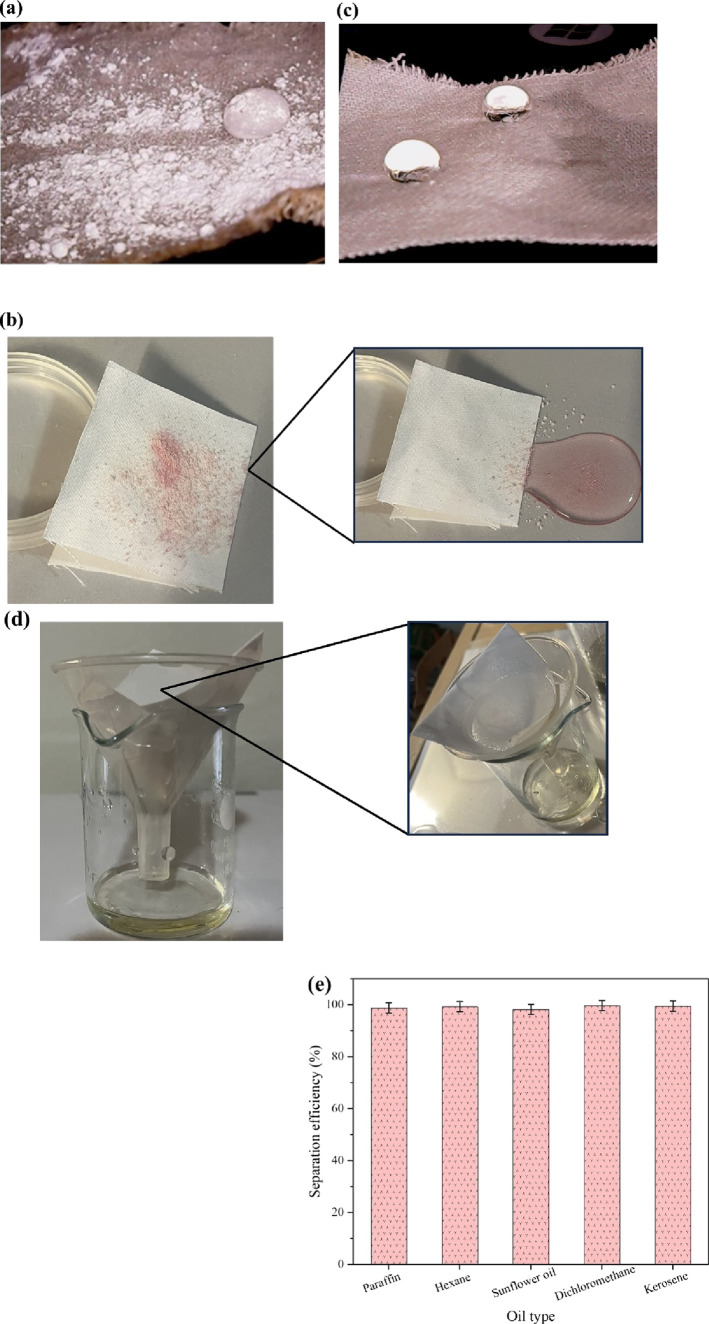
(a, b) Self-cleaning property, (c) super hydrophobicity and oleophilicity, (d) oil–water separation performance, and (e) separation efficiency of the Al_2_O_3_/MMT-PDMS coated fabric.

Self-cleaning properties allow the surface to repel water and prevent the adhesion of dirt, stains, and contaminants. Inspired by our previous studies,^21,47^ the self-cleaning property was evaluated through spreading a mixture of Al_2_O_3_/SiO_2_ and dye powder on the coated fabric as contaminant and sliding a water droplet throughout the surface. To this end, after placing the water droplet, the surface was tilted from 0°. The behavior of the droplet is shown in Figure 7a,b. The unique surface structure and chemistry of the as-prepared superhydrophobic fabric enable water droplets to form spherical shapes and easily roll off, carrying away any particles or impurities present on the fabric’s surface, which can be attributed to the reduced contact area between liquids and minimized adhesion forces resulting from the combination of micro- and nanoscale roughness. It can be concluded that the self-cleaning property of the fabric makes it ideal for use in marine environments, oil spill cleanup, and outdoor signage. Moreover, the fabric’s oleophobic/oleophilic behavior is assessed by placing an oil droplet on its surface, and the corresponding outcome is delineated in Figure 7c. As can be seen, the oil droplet is quickly absorbed by the coated fabric, which verifies the super oleophilic property and applicability for oil–water separation.

The oil/water separation performance of the as-prepared superhydrophobic fabrics was evaluated. Figure 7d depicts the performance of the coated fabric in the separation of sunflower oil/water. To this end, kerosene/water, dichloromethane/water, hexane/water, sunflower oil/water, and paraffin/water mixtures in 1:1 volumetric ratio were prepared and subjected to filtration using the coated fabric. To assess the performance of the coated fabric, it was fixed at the bottom of the tube, and the oil/water mixture was poured into the tube. Then the permeate was collected, and the oil/water separation efficiency was calculated using eq 1:^76^
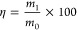
1where η is the separation efficiency and *m*_0_ and *m*_1_ are the mass of the initial and collected oil, respectively.

Upon contact with the oil/water mixture, the coated fabric rapidly absorbed the oil, confirming its highly selective sorption capabilities for the sorption of oil from water. However, despite its oil-absorbing properties, the fabric prevents the complete permeation of water, resulting in the water being retained above the superhydrophobic coated fabric. Figure 7 illustrates the separation efficiency of the different oil/water mixtures. It is evident that the separation efficiency (%) falls within the range of 98.2–99.7%. The slightly lower separation efficiencies observed for sunflower oil and paraffin (98.2 and 98.8%, respectively) can be attributed to their higher viscosity and complex composition,^50^ which pose challenges in the separation process. It is noteworthy to highlight that the evaluation of the separation efficiency of the superhydrophobic fabric for each oil/water mixture was conducted in triplicate. The reported values in this study represent the averages of these three independent measurements.

### 3.7 Chemical Stability

**Figure 8:**
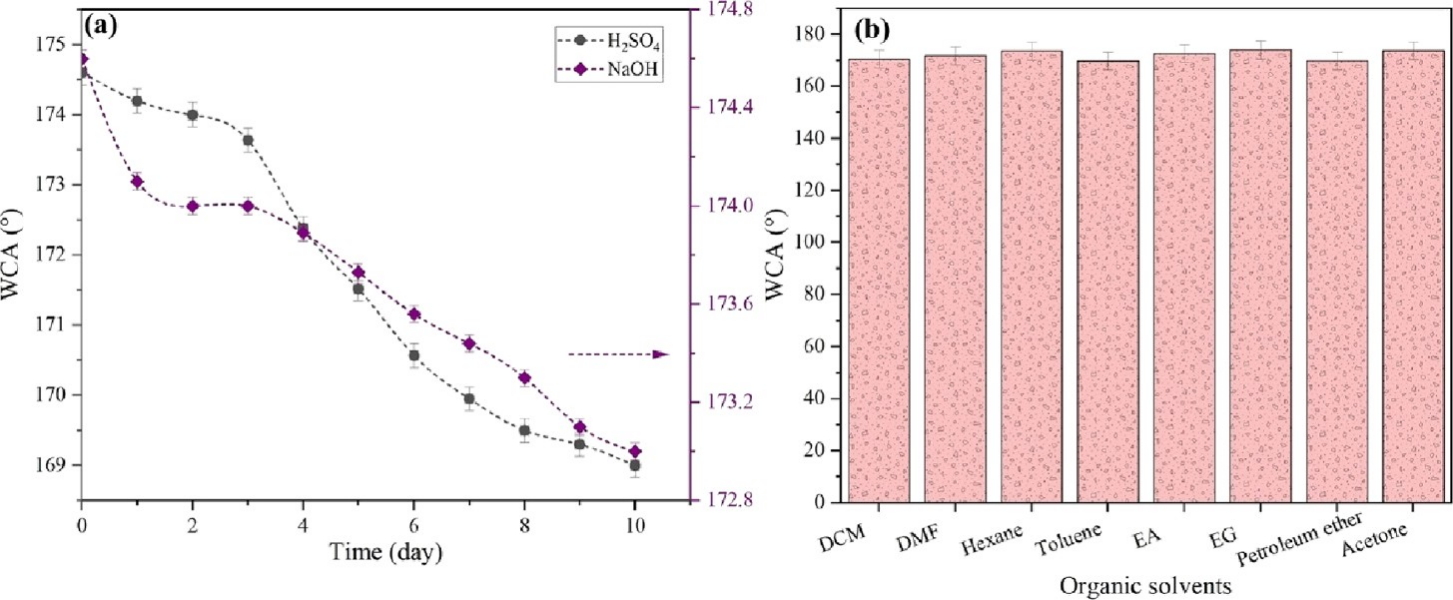
WCA of the coated fabric (a) as a function of immersion time in acidic (H_2_SO_4_, pH 2) and alkaline (NaOH, pH 12) media and (b) immersion in different organic solvents for 24 h.

Because of diverse applications of superhydrophobic surfaces, including transportation, architecture, and manufacturing, these surfaces may encounter aggressive chemicals, pollutants, or harsh conditions. The ability to withstand harsh and corrosive media without significant degradation or loss of water-repellent properties allows them to adapt to diverse environments while maintaining optimal performance. Also, it is crucial for practical applications to ensure long-lasting performance and durability. In the present study, the chemical stability of the coated fabric was evaluated through its immersion in strong acidic and alkaline media prepared using H_2_SO_4_ and NaOH, respectively. Based on the findings presented in Figure 8a, it is evident that the coated layer experienced an insignificant decline in WCA after immersion in acidic and alkaline media for 10 days. Specifically, a decrease of approximately 3.2 and 1% in WCA was observed for the coated layer exposed to acidic and alkaline environments, respectively. Following a 10-day immersion period, the coated layer maintained its superhydrophobic property, as evidenced by WCA > 150°. Thus, the Al_2_O_3_/MMT-PDMS coating exhibited promising stability in corrosive media. Meanwhile, to further assess the chemical stability of the as-prepared superhydrophobic fabric, it was subjected to immersion in various organic solvents, such as dichloromethane (DCM), dimethylformamide (DMF), hexane, toluene, ethyl acetate (EA), ethylene glycol (EG), petroleum ether, and acetone, for 24 h, and results are depicted in Figure 8b. It can be seen that throughout the 24 h immersion in organic solvents, the WCA > 150° of the coated fabric indicates the fabric’s stable and persistent superhydrophobicity.

### 3.8 Durability

The coated layer’s durability was evaluated through exposure to air for 1e month, ultrasonic washing for 12 h, and home laundering for 50 cycles. To evaluate washing resistance, durability was evaluated using the American Association of Textile Chemists & Colorists (AATCC) test method 61-2006 No. 2A, equivalent to five home laundering cycles.^56^

**Figure 9:**
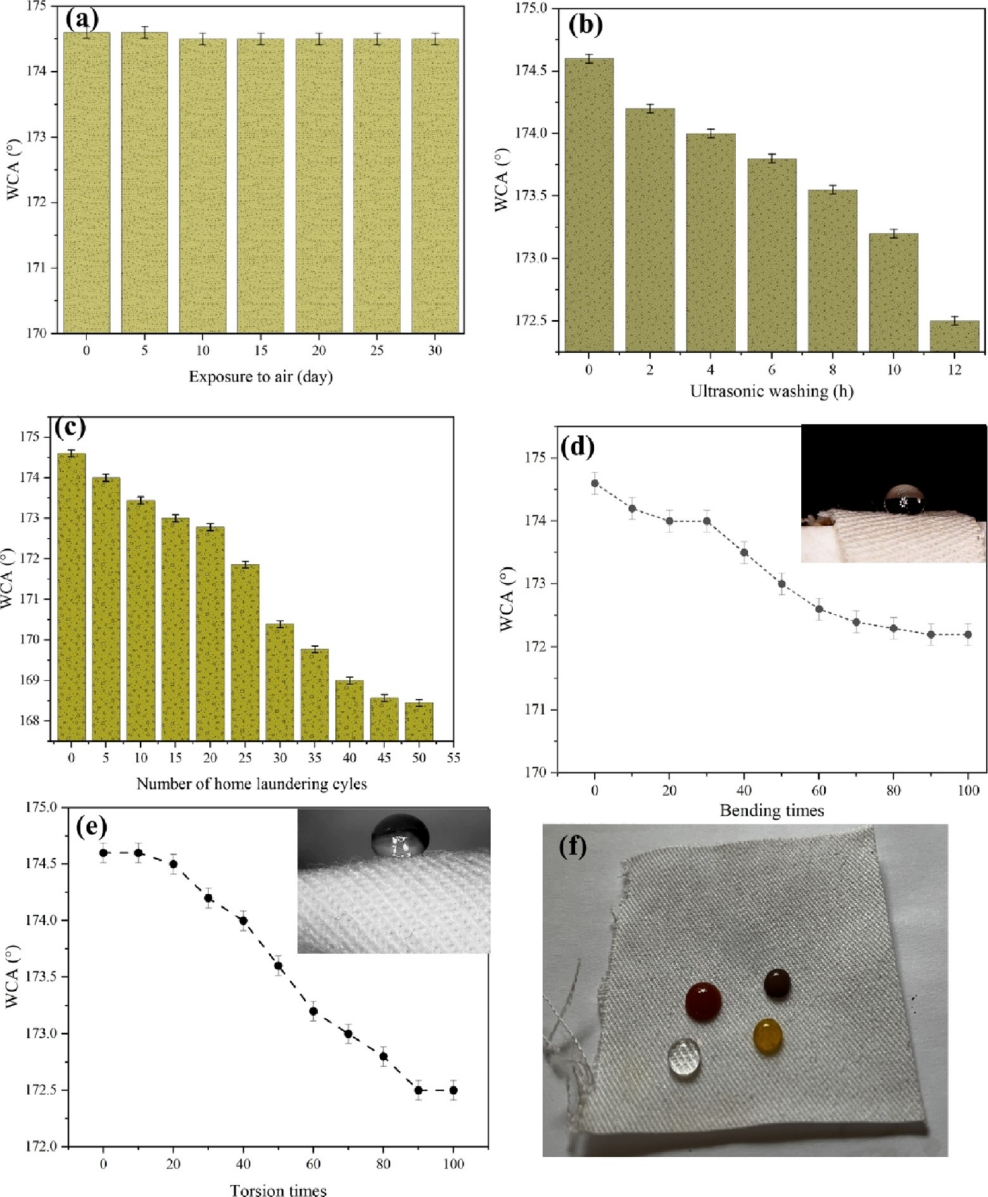
WCA of the coated fabric as a function of (a) exposure time to the air, (b) ultrasonic washing time, (c) home laundering cycles, (d) bending times, and (e) torsion times. (f) Photographs showing the spherical shape of water, coffee, ketchup, and colored water droplets on the coated fabric.

The durability of a superhydrophobic fabric against prolonged exposure to air refers to its ability to maintain the superhydrophobic properties and resist degradation in contact with air over time. This entails the maintenance of the fabric’s micro/nanostructured roughness surface, stability, and resistance of the surface coating against degradation; the accumulation of contaminants, including dust, dirt, or pollutants; and ultraviolet (UV) radiation from sunlight in terms of outdoor usage. Figure 9a depicts the wettability behavior of the coated layer exposed to the air for 1 month. WCA depicted an insignificant change as a function of time (dropped from 174.6 to 174.5°).

To assess the fabric’s resistance to mechanical stress generated by ultrasonic waves, the as-prepared fabric underwent ultrasonic washing, an effective method for eliminating dirt, stains, and contaminants. The superhydrophobic property of the fabric was then evaluated under these conditions. Figure 9 delineates the durability of the coated layer against ultrasonic washing. After 12 h of ultrasonic washing, a 1.2% decrease in WCA was observed. It can be verified that the superhydrophobic coating applied to the fabric was well-bonded and firmly adhered to the surface, effectively resisting detachment or damage during ultrasonic cleaning.

The durability of a superhydrophobic fabric against laundry washing refers to its ability to withstand mechanical stress, detergents, and repeated washing cycles typically involved in standard laundry processes without significant degradation or loss of its superhydrophobic properties. The wetting behavior of the coated layer as a function of washing cycles is depicted in Figure 9c. The WCA of the droplet placed on the coated fabric was evaluated after each washing cycle, and it can be verified that 3.5% of the WCA was diminished after 50 cycles of home laundering. Regarding the results (WCA = 168.4 in the 50th cycle), it can be concluded that the coated layer maintained its integrity and adhered to the surface against the agitation and rubbing forces generated during washing, common laundry detergents, and repeated washing cycles.

Figure 9d,e delineates the analysis of the variation in WCA against bending/releasing and twisting/releasing tests, respectively. The inset figures within these plots offer a visual representation of the overall shape of the water droplets placed on the fabric after undergoing bending and twisting. Results reveal that, after subjecting the coated fabric to 100 cycles of bending/releasing, there was an insignificant decrease in WCA by ∼2%. Similarly, in the case of twisting/releasing tests, the WCA exhibited a reduction of ∼2% after 100 cycles. This subtle change underscores the remarkable stability and flexibility of the coated layer under repeated bending and twisting cycles. This observation also suggests that the structural integrity and hydrophobic properties of the coated layer remain largely unaffected. The minimal shift in water contact angle reflects the robust nature of the coating, emphasizing its resilience and ability to maintain hydrophobic performance.

Figure 9f depicts spherical water, coffee, ketchup, and colored droplets placed at the surface of the coated fabric subjected to multiple cycles of bending and twisting. Notably, the coated layer remarkably retains its super hydrophobic property. Similarly, the coffee, ketchup, and colored droplets exhibit minimal spreading or absorption, verifying the resistance to various liquids.

### 3.9 Mechanical Resistance

**Figure 10:**
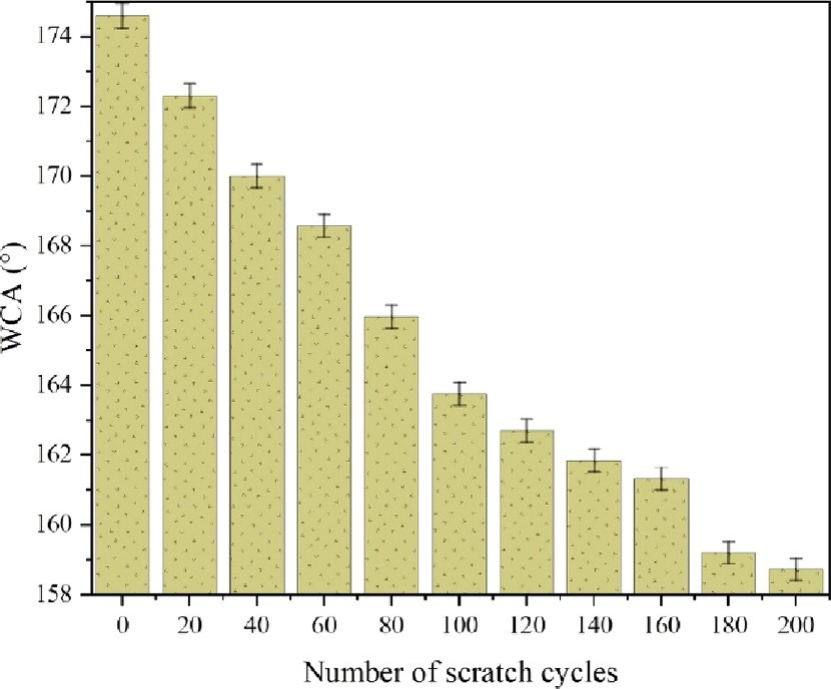
WCA of the coated fabric as a function of the scratch cycles.

The mechanical resistance of a superhydrophobic fabric pertains to its capacity to endure mechanical stress, including stretching, bending, and abrasion, without sustaining significant damage or compromising its superhydrophobic properties. In this paper, to evaluate the mechanical resistance of the coated layer and compare the behavior of the layer with previous studies,^21,47^ the coated fabric was positioned on an 800 mesh SiC sandpaper, and it was subjected to 200 back-and-forth dragging cycles while applying a 200 g weight on the fabric. Figure 10 depicts the WCA of the coated fabric after each abrasion cycle. Results showed that a ∼9.1% drop in WCA was observed after 200 cycles of scratching (WCA plummeted from 174.6 to 158.7° in the 200th cycle). The formation of cross-linking and grafting structures via silanol groups confirms the robustness of the nanostructure, providing protection against easy damage or degradation.^77^

The fabric retained its super hydrophobicity, and the coated layer displayed promising resistance against scratching. The results indicated that the coated layer exhibited substantial strength and resilience to abrasion and wear, as experienced during regular usage, including friction and contact with rough surfaces. This notable durability can be attributed to the highly cross-linked network formed on the fabric surface by the combination of PDMS, DBTDL, TEOS, and the Al_2_O_3_/MMT nanocomposite.

### 3.10 Thermal Resistance

**Figure 11:**
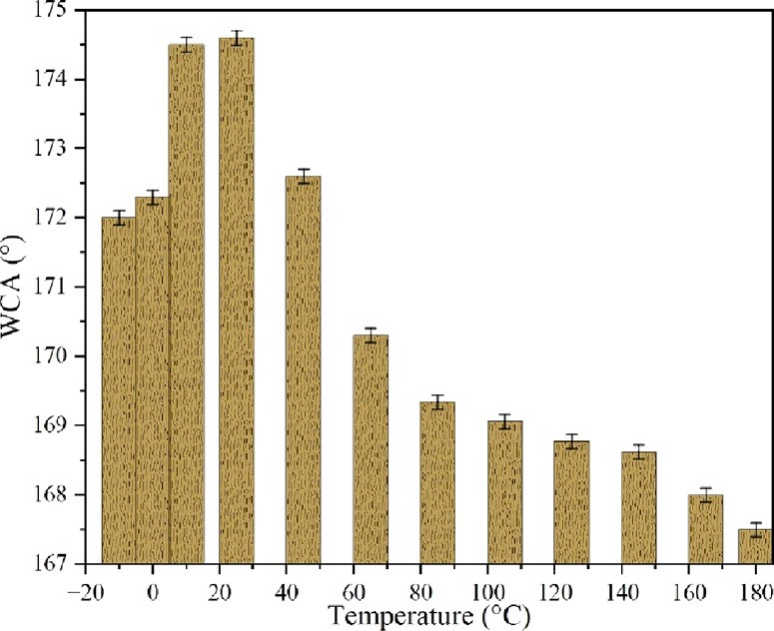
WCA of the coated fabric as a function of the treatment temperature.

The thermal resistance of a superhydrophobic fabric is its capability to endure elevated temperatures without experiencing notable degradation, loss, or alterations in its structure or properties under thermal stress. To assess the thermal resistance property, the coated fabric underwent thermal treatment at various temperatures ranging from −10 up to 180 °C for a duration of 12 h. Figure 11 verifies the WCA behavior of the treated fabric. Regarding the results, ∼1.5% WCA decline was observed for temperatures <25 °C, whereas ∼4.1% of WCA was dropped for higher temperatures up to 180 °C. Superhydrophobicity of the fabric was maintained at different temperatures. Compared to the previous studies,^21,47^ the coated layer showed better thermal resistance in the −10 to 180 °C range.

### 3.11 Comparison with Previous Studies

This study focuses on the formation of a long-lasting superhydrophobic layer on cotton fabric. This was achieved by the incorporation of the Al_2_O_3_/MMT nanocomposite into the surface followed by spray coating of the PDMS layer. Table 3 summarizes the characteristics of some of the recent superhydrophobic fabrics reported in previous research. It is noteworthy that some of the prior studies have primarily centered on the utilization of expensive and environmentally harmful long-chained and fluorinated precursors. This not only poses economic challenges but also raises concerns about the potential adverse effects on the environment and human health.^47^ Furthermore, certain investigations have employed multiple precursors, multistep processes, or harsh preparation conditions^47^ such as etching. The multi-step and time-consuming nature of such processes can be impractical, particularly when considering coating materials that are thermosensitive, such as fabrics.^51^ The need for mild preparation conditions becomes crucial to ensure that their integrity and functionality are preserved during the coating process. Therefore, exploring alternative methods and materials that address these limitations is essential for the advancement and applicability of superhydrophobic surfaces in diverse contexts. In this study, Al_2_O_3_, MMT, and PDMS were used as affordable, abundant, and environmentally friendly materials to form a durable and self-cleaning superhydrophobic layer on the fabric, which not only enhanced the fabric’s longevity but also aligned with our commitment to eco-friendly practices. Moreover, the spray coating method offers a straightforward and cost-effective solution suitable for large-scale applications. This method ensures practicality in implementation and economic viability. Besides, the results demonstrate that both the water contact angle and water sliding angle exceed those reported in previous studies. This signifies a substantial improvement in achieving superior water-repellent properties.

**Table 3:** Summary of Some Recent Superhydrophobic Fabrics Reported in Previous Studies

precursors	method	WCA (°)	WSA (°)	ref
octahedral Cu-1,3,5-benzenetricarboxylic acid (CuBTC) crystals, 1*H*,1*H*,2*H*,2*H*-perfluorooctyltriethoxysilane, triethoxyoctylsilane	layer by layer deposition	168.4	1.8	(78)
gellable fluorinated block copolymer poly(dodecafluoroheptyl methacrylate)-*block*-poly(3-(triethoxysilyl)propyl methacrylate) (PDFMA-*b*-PTEPM), SiO_2_ nanoparticles	pip-coating (two-step sol–gel method)	160.2	<10	(77)
polycaprolactone, fluorinated phenyl polysesquisiloxane and propyl acetate emulsion, vinegar–acrylic emulsion	electrostatic spraying	<160	<1	(79)
methyltrimethoxysilane (MTMS), polydimethylsiloxane (PDMS), peanut shell powder, sodium chlorite (NaClO_2_), sodium carbonate (NaCO_3_), sodium bicarbonate (NaHCO_3_), 2,2,6,6-tetramethylpiperidine oxide (TEMPO),	spray coating	160		(80)
single-walled carbon nanotubes (SWCNTs), 1*H*,1*H*,2*H*,2*H*-perfluorooctyltriethoxysilane (POTS)	dry deposition and solution immersion	163.3		(81)
benzoin dimethyl ether, sulfhydryl modified rosin acid, octenyl-POSS, SiO_2_ nanoparticles	spray coating	168.6		(82)
SiO_2_ nanoparticles, 2,2,6,6-tetramethylpiperidinyl-1-oxyl (TEMPO), isocyanate (IPDI), polydimethylsiloxane (PDMS)	spray coating and immersion	158.6	∼7	(83)
SiO_2_ nanoparticles, aluminum phosphate, hexadecyltrimethoxysilane (HDTMS)	spray coating	151.8	<5	(84)
Al_2_O_3_ montmorillonite (MMT), dodecyltrimethoxysilane (DTMS), tetraethyl orthosilicate (TEOS), polydimethylsiloxane (PDMS)	spray coating	174.6	<5	present study

## 4 Conclusions

The current study depicts the successful fabrication of a superhydrophobic fabric using the Al_2_O_3_/MMT nanocomposite and PDMS polymer. The incorporation of the Al_2_O_3_/MMT nanocomposite has significantly increased the surface roughness of the fabric, whereas the application of the PDMS polymer has effectively reduced the surface tension, leading to the development of the desired superhydrophobic properties. This combination has resulted in a fabric with exceptional water repellency, as demonstrated by WCA = 174.6° and WSA < 5°. As an efficient, time-saving, cost-effective, and scalable method, the spray coating procedure was utilized to apply the superhydrophobic layer onto the fabric surface. Regarding several gaps in the fabrication of superhydrophobic coatings, including challenges related to complex chemical composition and the use of fluorine-containing precursors, as well as the vulnerability of fragile constructions, this work makes new contributions to the existing body of knowledge by (1) selecting MMT with a layered structure as a substrate to enhance the nano/micro-roughness of the fabric surface through the growth of Al_2_O_3_ nanoparticles and (2) utilizing an environmentally friendly and cost-effective PDMS polymer to reduce surface tension.

The as-prepared superhydrophobic fabric exhibited desirable properties such as self-cleaning capabilities that resulted in cleaning dust and dirt on the surface, promising chemical stability (3.2 and 1% loss in WCA after 10 days of immersion in highly acidic and alkaline media) and WCA > 160° in case of 24 h immersion in various organic solvents, excellent durability against exposure to air (WCA = 174.5° after 1 month), resistance to washing (WCA = 172.5° after 12 h of ultrasonic washing and WCA = 168.45° after 50 cycles of home laundry), mechanical abrasion resistance (WCA = 158.7° after 200 cycles of abrasion), and thermal resistance (<4.1% of WCA loss in the −10 to 180 °C range). Additionally, the fabric demonstrated the promising ability to separate oil from water (>99% oil (paraffin, hexane, sunflower oil, dichloromethane, and kerosene)–water separation).

The developed superhydrophobic fabric holds significant potential for applications in various fields including protective clothing, outdoor gear, medical textiles, and sportswear. Its water-repellent nature, self-cleaning properties, and durability make it an ideal choice for use in demanding and challenging environments.

## Supporting Information Available

Video showing the superhydrophobic behavior of the Al_2_O_3_/MMT-PDMS coated fabric in contact with a water droplet (AVI)

## Supplementary Material

la3c02325_si_001.avi
